# Liquid metal droplet shuttling in a microchannel toward a single line multiplexer with multiple sensors

**DOI:** 10.1038/s41598-022-08611-4

**Published:** 2022-03-16

**Authors:** Ayano Shimizu, Yugo Kakehi, Shinji Bono, Satoshi Konishi

**Affiliations:** 1 Graduate Course of Science and Engineering, Ritsumeikan University, Kusatsu, 525-8577 Japan; 2 Department of Mechanical Engineering, College of Science and Engineering, Ritsumeikan University, Kusatsu, 525-8577 Japan; 3 Ritsumeikan Global Innovation Research Organization, Ritsumeikan University, Kusatsu, 525-8577 Japan

**Keywords:** Electronic devices, Fluidics, Fluids, Metals and alloys

## Abstract

Multiple sensors and actuators integrated in a small space, especially an elongated thin structure, require equivalent number of signal lines between microdevices, but there is limited space for signal wires. Thus, we propose a mechanism using a single microchannel where a liquid metal droplet moves and shuttles. A shuttling droplet switches multiple terminals of signal lines along a microchannel based on a traditional switching mechanism using a liquid metal droplet. Electrically conductive gallium alloy liquid metals (Galinstan) can flow in a microchannel due to their fluidity. The terminals consist of opposing electrode pairs in a microchannel. A change in a variable impedance connected to a terminal as a pseudo sensor can be read when a droplet flows in and connects electrode pairs. This paper presents switching and addressing objective terminals of chromium electrodes by a shuttling conductive droplet (500 µm in diameter and 10 mm long) in a microchannel (500 µm in diameter and 100 mm long). A demonstrated simple mechanism enables communication between multiple microdevices along a microchannel. We anticipate wide application of proposed mechanism toward a multiplexer, especially in microfluidic devices because of the advantages of utilizing microchannels as common microstructures for both microdevices and signal lines.

## Introduction

Integration technology of sensors and actuators has progressed by micro electro mechanical systems (MEMS) technology based on integrated circuits technology. Small tools functionalized with multiple sensors and actuators expand possibilities of MEMS in various fields, such as bio and medical applications. Catheters and endoscopes have been developed for minimally invasive medicine, which requires performance in narrow and complex spaces, such as inside of a human body. An industrial endoscope is generally useful in similar narrow and complex spaces in machines and in the rubble of disaster sites, in addition to in vivo medical procedures. An elongated thin structure for catheters and endoscopes is equipped with multiple actuators to drive and move the devices with multiple degrees of freedom. It has become important to equip endoscopes with sensors to recognize their own conditions, such as deformation and attitude^1,2^. There is, however, a limitation in the space available for additional devices within such a thin structure. Moreover, sensors and actuators arranged along an elongated structure require signal transmission along a central control site. It is not efficient to separately wire a central control site to all individual devices because the number of wires increases too much. There is limited space available for wiring and local signal processing, as catheters and endoscopes decrease in diameter. Several studies have reported reduction in the number of wires for catheter sensors and actuators^3,4^. Multiple shape memory alloys (SMAs) for articulated miniature devices have been actuated and controlled using a minimum number of connecting wires^3^. SMAs arranged in a matrix were switched by two solid-state switches. An active multilink catheter integrated with CMOS interface circuits for communication and control was reported^4^. The interface circuits used three common lead wires to address and control the selected actuators in the active catheter.

A multiplexer is often used to output a selected signal among multiple signals, which requires a single line for the output^5,6^. In addition to electrical multiplexers, optical multiplexers for switching optical signals have been recently reported^7^. Techniques for discriminating multiple signals using light have also been reported^8,9^. This technique enables switching of multiple signals, even at nanoscale sizes, and is suitable for narrow spaces because of the fiber array. A multiplexed transmission system based on frequency-division multiple access using surface acoustic wave filters was studied to reduce the number of electrical signal wires for individual switching devices and circuits^10^. Neural interfaces, such as the brain, spinal cord, and peripheral nerves, require parallel signal recording and processing by minimally invasive devices. Neural probes with functions for the parallel readout of 144 recording sites were reported^11^. The microprobe was 70 μm wide, 50 μm thick, and 5.6/11.5 mm long. The designed architecture for the neural probes could avoid the global routing of neural signals by connecting with a digital four-wire interface.

This study proposes channels or tubes for both electrical signal transmission and fluidic supply in long and thin devices. We are interested in liquid metal for conductive materials for electrical functions, such as electrodes and wires. It is possible to introduce liquid metal into channels and tubes due to the fluidity and electrical conductivity of liquid metal. The combination of liquid metal and microfluidic technologies using low-cost microstructures has allowed to develop micro-fluidically tunable electrical devices, such as tunable capacitors, switches, and antennas^12^. The channels and tubes filled with conductive material work as electrodes and electrical wires. It has been reported that liquid metal can be filled into complex channels, where the vacuum-filling is also effective^13^. Wires and flexible devices using flexible microfluidic channels filled with liquid metal have been reported^14–16^. Several switching devices use liquid metal and alloys, such as mercury and Galinstan (eutectic gallium indium stannum)^17,18^. It was reported that the electrical capacitance was tuned by moving mercury droplets between electrode pairs. Mercury has been traditionally studied; however, its use has been halted due to its high toxicity and environmental influence. Galinstan, with flowability and conductivity, is attracting attention as an alternative to mercury because of its low toxicity^19^. Galinstan is attractive as a substitute mercury for microdevices in need of biocompatibility, such as wearable devices. Galinstan is easily oxidized in the atmosphere. The oxide layer growing on the surface of Galinstan causes adhesive problems when Galinstan is introduced into a fluidic channel. The oxide layer adheres to the inside wall of the fluidic channel and prevents Galinstan from moving smoothly^20^. It was reported that introducing a liquid such as water into the channel along with Galinstan can prevent the oxide film from adhering to the channel^21^. As a result, Galinstan can move smoothly in the channel. It has been also reported that motion of liquid metal is controlled by applying voltage in the chancel^22^. The behavior of liquid metal droplet in convection has been examined for transportation of liquid metal droplet in a fluid channel^23^. It has also been presented that local electrical capacitance could be modulated by sandwiching the liquid-introduced channel between electrode pairs and smoothly moving Galinstan droplets into the channel^24^.

In this paper, we design liquid metal droplet shuttling in a microchannel toward a single line multiplexer with multiple sensors, which can transmit signals between multiple sensors arranged in a long and thin structure with limited space. Presented method expands switching function of traditional single switches using motion of a liquid metal droplet to multiple switching along a microchannel. The designed mechanism employs a liquid metal droplet moving in a fluidic microchannel. The moving droplet in the channel switches multiterminal electrodes along a channel and reads signals from microsensors connected to the terminal electrodes. Elemental components are the only two electrical signal lines along with one fluidic channel for the mechanism using a shuttle of Galinstan droplets. Multiterminal electrodes composed of opposing electrode pairs are fabricated in the channel. Sensors are connected to one of the electrode pairs. A variable resistance is connected to one of electrode pairs to demonstrate microsensors in this study. Liquid metal droplet flows in the channel and connects electrode pairs of an objective terminal. The sensor signal can be read and transmitted via signal lines when the droplet connects the terminal. The position of a droplet can be detected as capacitance change of the terminal, whereas the value of the resistive sensor can be read and acquired as the resistance change of the terminal. This paper presents switching and addressing objective terminals connected to variable impedances by a shuttling Galinstan droplet.

## Results

**Figure 1:**
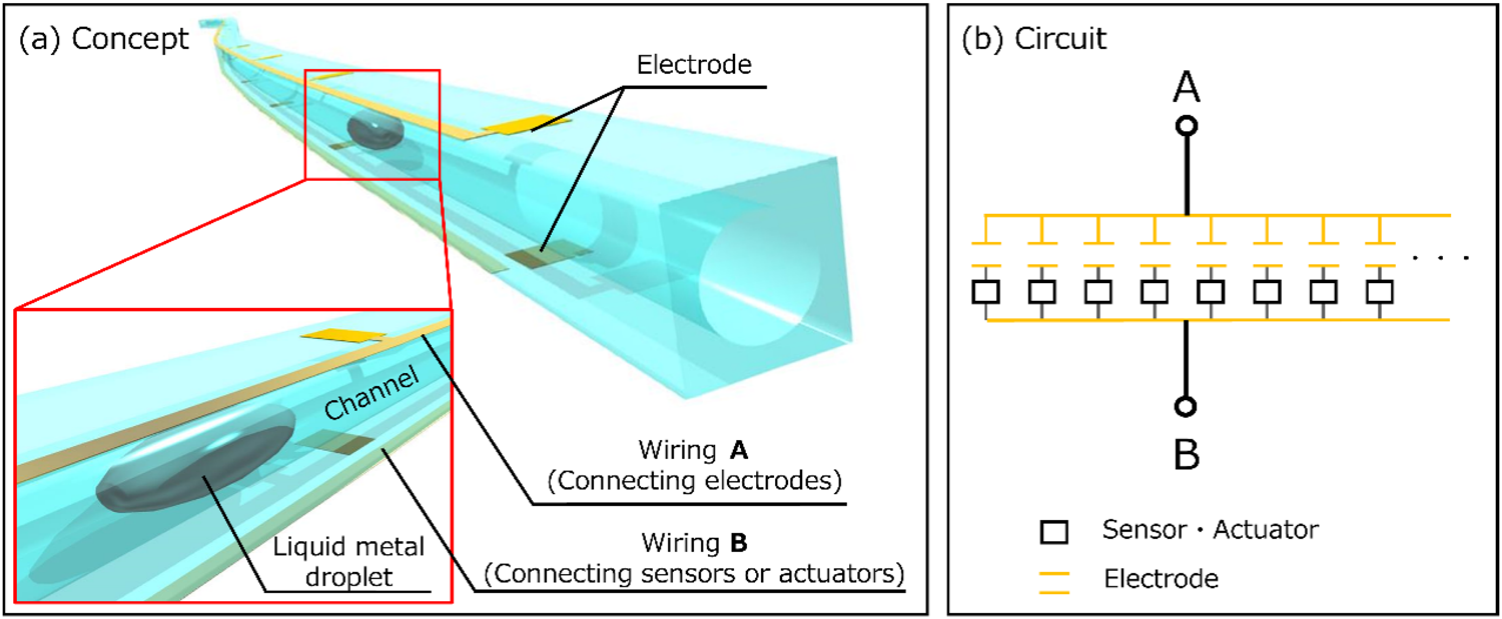
Overview of the new multiplexer. (**a**) Perspective schematic view of the conceptual multiplexer, which has a long microchannel structure. Shade3D Basic ver. 17.0.0 8 (https://shade3d.jp/en/) was used to create the images. (**b**) Equivalent circuit diagram of the multiplexer connected to sensors or actuators.

Figure 1a shows the concept of the proposed multiplexer. It has a thin and long microchannel structure. To prevent a Galinstan droplet from sticking to the microchannel wall, we introduce liquid such as silicone oil with a Galinstan droplet into the microchannel. We can control the position of the Galinstan droplet by applying pressure to the end of the microchannel. The microchannel of this multiplexer is sandwiched between electrodes. The top wiring A and the bottom wiring B connect with the electrode and the sensors and actuators, respectively. Figure 1b shows the equivalent circuit diagram. Capacitor parts represent electrodes. Sensors and actuators are connected to both the electrodes at the bottom wiring B. The two wires A and B are connected to a measuring device or a transmitter so that signals can be sent to and received from several sensors and actuators. When a Galinstan droplet passes between these electrodes, the capacitance changes. When a Galinstan droplet lodges between the electrodes, it is possible to obtain the signal value of the sensor actuator connected to the electrode pair.

**Figure 2:**
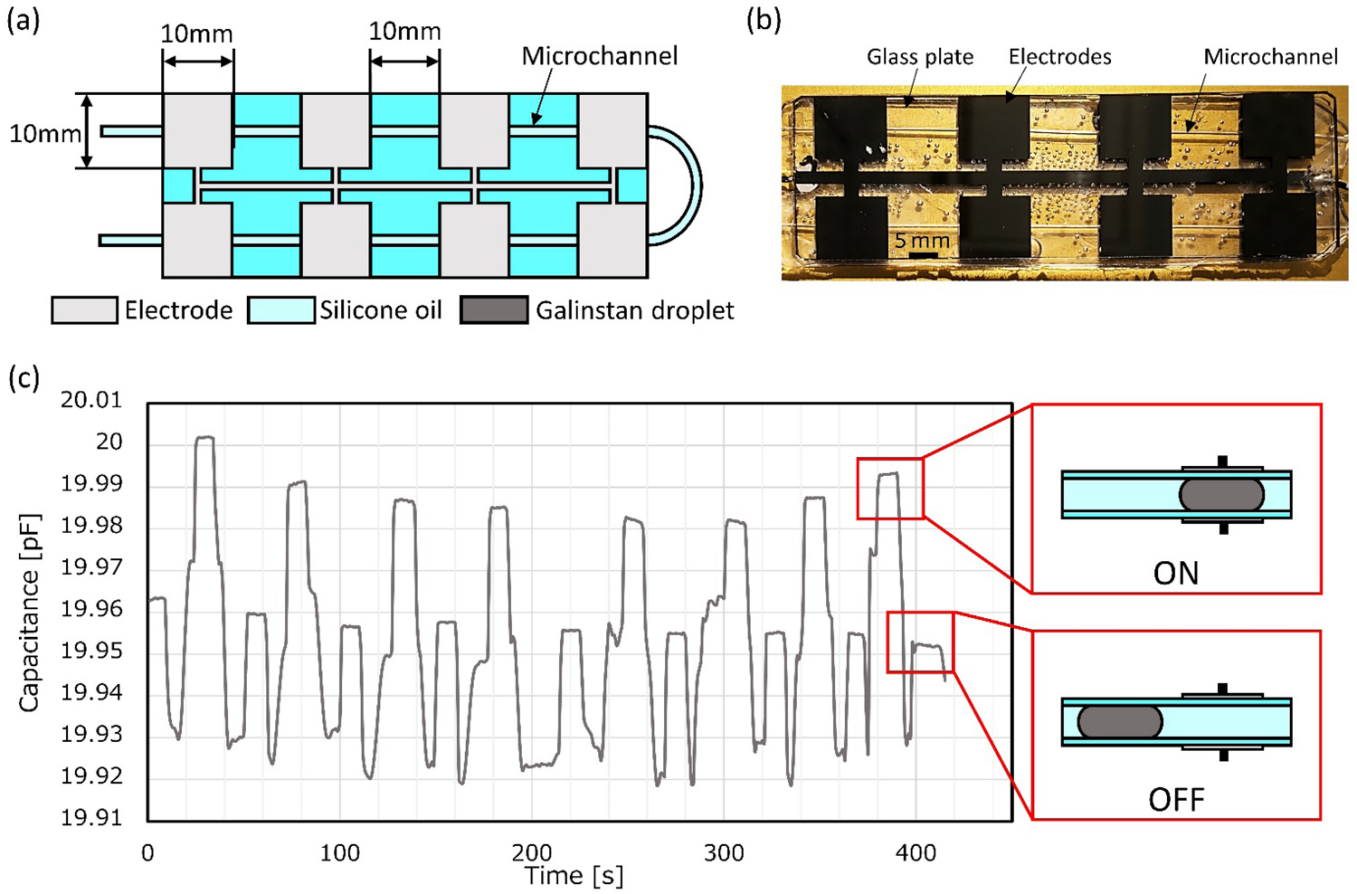
Array of electrodes for measuring the capacitance. (**a**) Top view of the 2^3^ electrode pair and microchannel sandwiched by the electrodes. (**b**) Top view picture of the 23 electrode and the microchannel. The microchannel is sandwiched between the glass plates. (**c**) Capacitance change when LMD passes between electrodes.

Previous work reported that the insertion of Galinstan droplets increases the capacitance of the pair of electrodes^24^. Then, we integrated 2^3^ pairs of electrodes into the microchannel and switched the capacitance of serially connected electrode pairs using the spatial control of Galinstan droplets. Figure 2 shows the change in capacitance of a microchannel sandwiched between 2^3^ pairs of electrode arrays through which the Galinstan droplet passes. Figure 2a shows the top view of the device and Fig. 2b shows a prototyped test structure. The microchannel is sandwiched between hard glass plates. We inject Galinstan droplets into a round microchannel with an inner diameter of 0.5 mm and a Galinstan droplet of a length of 5 mm. Here, we aim to show that the spatial control of Galinstan droplets can realize the selective acquisition of signals. Thus, for simple and stable measurement, we used thin Cr electrodes patterned on glass substrates. The gap between the electrodes is 1 mm. Both the top and bottom electrodes are connected to an LCR meter. To control the position of the Galinstan droplet, we apply pressure to the microchannel with a syringe. Figure 2c shows the capacitance when the Galinstan droplet is between each pair of electrodes. While we transported Galinstan droplets in the microchannel, flexible microchannel and the electrodes on a hard substrate contacted with each other without any disintegration. The capacitance increases as the Galinstan droplet enters the space between the electrodes and decreases when it leaves the space between the electrodes. We found that the insertion of the Galinstan droplet can switch the capacitance of the pair of electrodes selectively.

**Figure 3:**
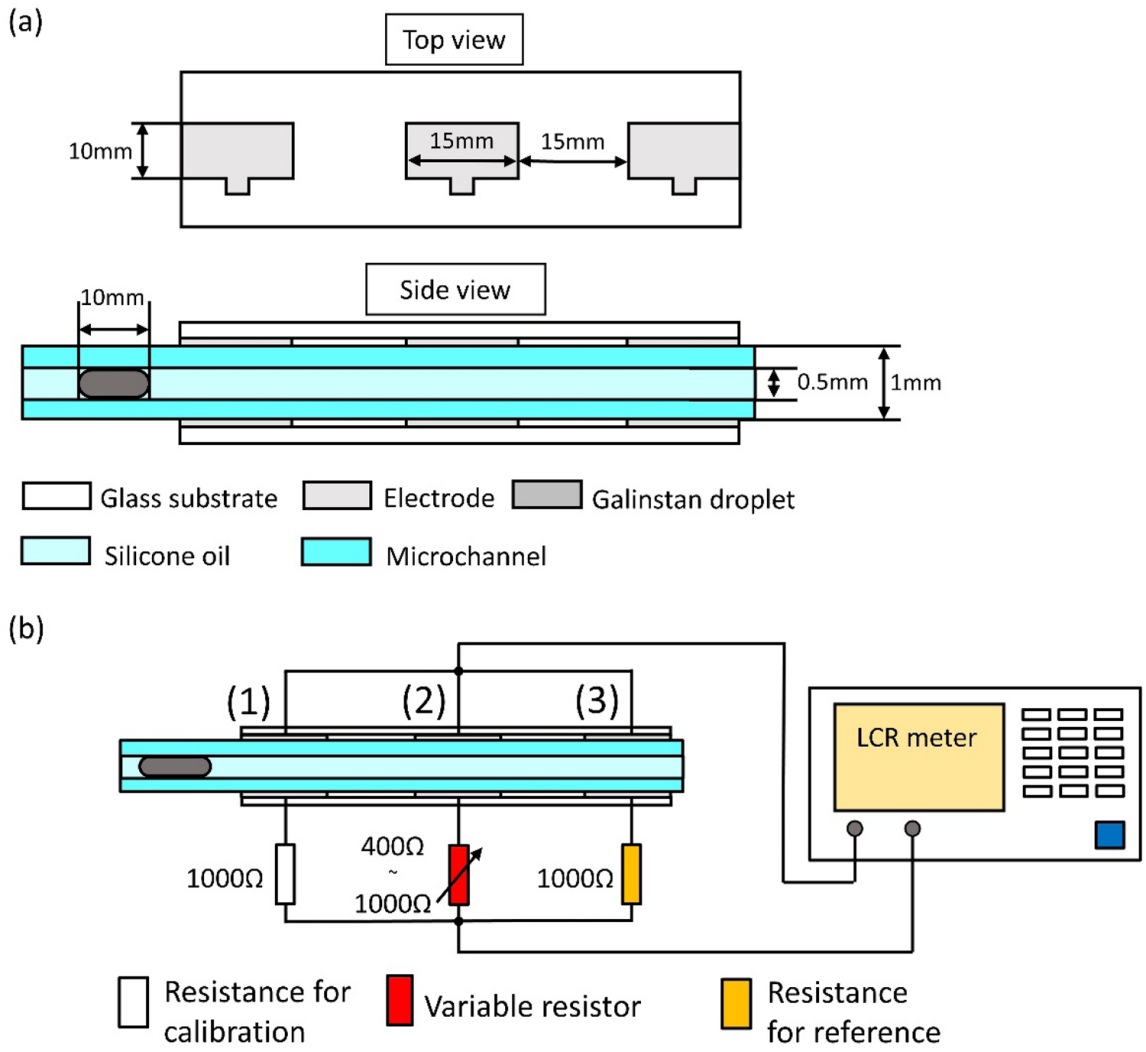
Experimental system for impedance measurement using model sensor. (**a**) The top view and the side view of multiplexer dimensions used in the experiment. (**b**) Circuit diagram of the experimental system.

Then, we connected sensors and the electrodes and transported the Galinstan droplets for the selective acquisition of signal. Using a model sensor, we measure the signal values of the sensor. We pattern three electrodes on a glass substrate, as shown in Fig. 3a. To stabilize the capacitance, we design the size of electrodes larger than the diameter of the microchannel (10 mm × 15 mm). The microchannel is filled with silicon oil to prevent Galinstan droplets from sticking to the microchannel wall. The inner diameter of the microchannel is Φ 0.5 mm and the gap between electrodes is 1 mm. The total length of the Galinstan droplet is 10 mm. If the length of the Galinstan droplet is larger than the interval of the neighboring pair of electrodes, the Galinstan droplet crosstalks between the electrodes. Therefore, the length of the Galinstan droplet must be smaller than the interval of 15 mm between adjacent electrode pairs.

In this experiment, we measure the signal of semiconductor strain gauges whose resistance changes. The initial resistance *R*_0_ of the semiconductor strain gauge and the gauge factor *K* are assumed to be 1000 Ω and approximately 200, respectively. In typical measurements, the strain of the semiconductor strain gauge should change by 0.1%. The amount of change in the resistance of the strain gauge can be obtained as *ΔR*/*R*_0_ = *K* × *ε*; therefore, ∆*R* is 200 Ω. Therefore, in this experiment, we investigate the signal from a model sensor in terms of the change in resistance of the model sensor. We show the experimental setup in Fig. 3b. As model sensors, resistance elements are connected to each of the three electrodes, and the impedance is measured. We connect 1000 Ω resistances to electrode (1) and electrode (3) for calibration and for the control experiment, respectively. Conversely, we connect a variable resistor to electrode (2) as a model sensor. We connect the upper electrode and the resistor element to an LCR meter; the voltage and frequency of the impedance measurement are 1 V and 1 MHz, respectively. In the experiment described above, we measure the impedance of the model device at the position of the Galinstan droplet and check if the signal from the model sensor can be extracted from the synthesized impedance. To obtain the signal independent of the position of the Galinstan droplet, we perform a control experiment. In the control experiment, we measure the synthesized impedance concerning the variable resistor when the Galinstan droplet is at electrode (3).

**Figure 4:**
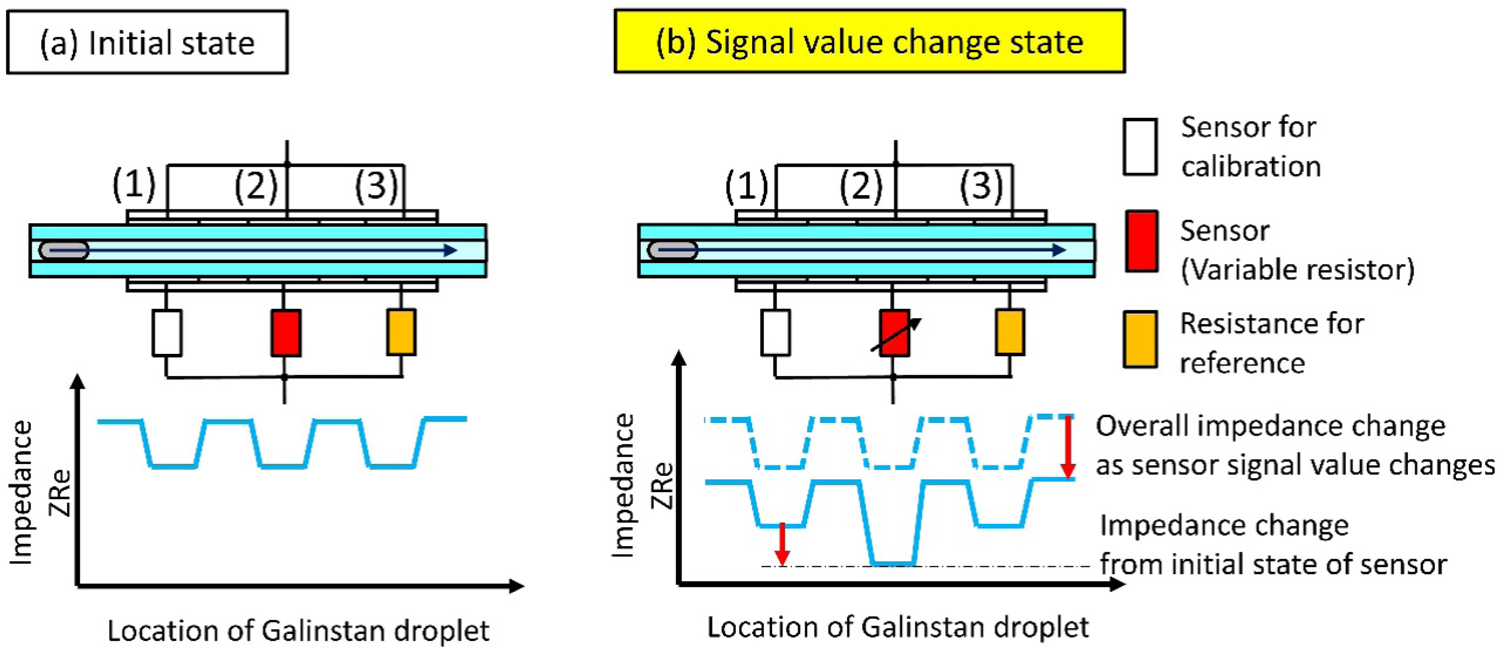
The need for a calibration sensor. (**a**) The impedance in the initial state when the Galinstan droplet moves from electrode (1) to electrode (3). (**b**) The impedance when the resistance of the valuable resistor decreases.

Let us discuss the behavior of the impedance when a Galinstan droplet moves from electrodes (1) to (3). When the resistances connected to each electrode are in the initial state (Fig. 4a), the impedance value depends on whether the Galinstan droplet is sandwiched by the electrode. In other words, the impedance is independent of electrodes (1), (2), and (3) because the resistances connected to the electrodes are the same. If the resistance of the variable resistor connected to electrode (2) is decreased, the impedance changes from the initial value as shown in Fig. 4b. Since our model device is a parallel circuit, the impedance decreases even if the Galinstan droplet is not sandwiched by electrode (3) connected to the unchanged resistance. Therefore, the difference between the initial impedance and the impedance when the resistance of the variable resistor changes with the Galinstan droplet sandwiched by electrode (2) does not allow us to obtain an independent signal change. It is necessary to connect a sensor for calibration, which indicates the initial state of the sensor, to the other electrode separately from the sensor. We calculated the rate of the change of the impedance between the Galinstan droplet sandwiched by electrode (1) connected to the resistance for calibration and when there is a Galinstan droplet sandwiched by electrodes (2) connected to the sensor. By examining the rate of change, it is possible to determine how much the sensor signal has changed.

**Figure 5:**
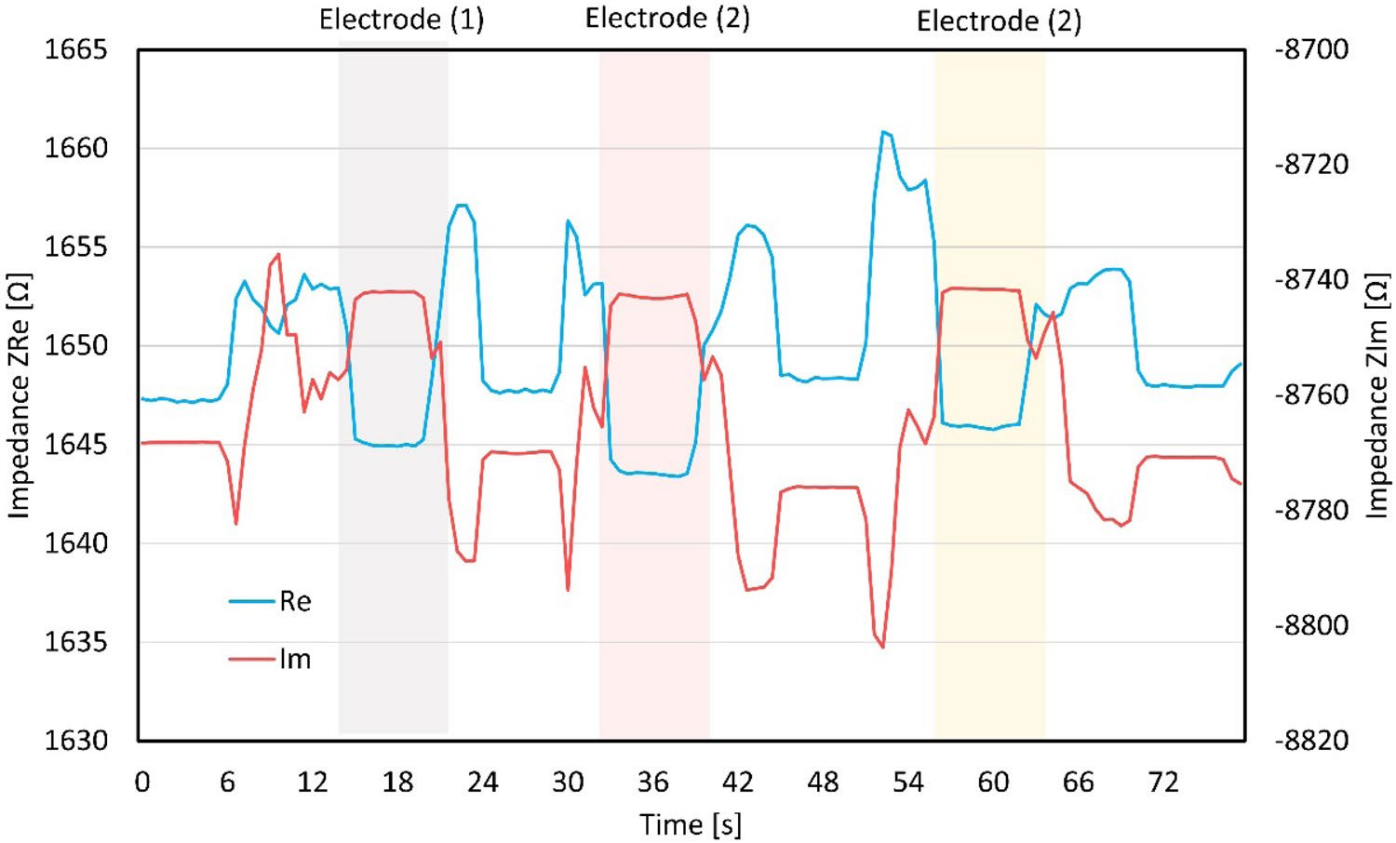
Dependence of the real and imaginary impedances on the Galinstan position with 1000 Ω of the variable resistor. The impedance changes when the Galinstan droplet is sandwiched by the electrodes.

Using an LCR meter, we measure the real and imaginary parts of the impedance. The measured impedance is plotted in Fig. 5. The value of the variable resistor of electrode (2) in Fig. 5 is 1000 Ω. We find that the impedance of the imaginary part *Z*_*Im*_ abruptly increases when a Galinstan droplet is positioned between the electrodes. This means that it is possible to switch between the ON state, where a sensor signal is obtained, and the OFF state, where no signal value is obtained; thus, it is possible to determine which electrode contains a Galinstan droplet. On the other hand, the impedance of the real part decreases when a Galinstan droplet enters the electrode. When Galinstan droplets are present in the electrode, the impedance remains constant (Fig. 5). For the measurement of the resistance connected to each electrode, the data of the real impedance *Z*_*Re*_ are taken with a Galinstan droplet in the respective electrode.

**Figure 6:**
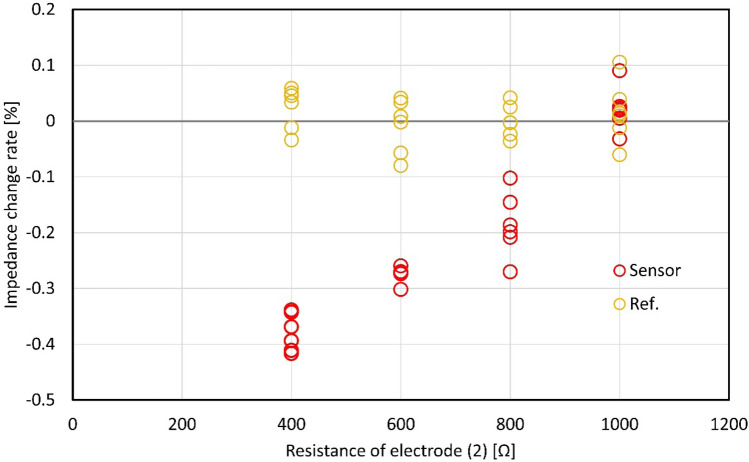
Impedance change rate of each resistance to the resistance for calibration. The rate of change of impedance of sensor signal changes when the resistance of the variable resistor connected to the electrode (2) changes. On the other hand, the rate of change of impedance of reference signal does not change regardless of the resistance value of the variable resistor.

The impedance is measured by changing the resistance of the variable resistor connected to electrode (2) to 1000 Ω, 800 Ω, 600 Ω and 400 Ω. The impedances when there is a Galinstan droplet at electrode (1), electrode (2), and electrode (3) are *Z*_1_, *Z*_2_*,* and *Z*_3_, respectively. The impedance change rate $\overline{{Z }_{2}}=({Z}_{2}-{Z}_{1})/{Z}_{1}$ of electrode (2) relative to electrode (1) is measured when a Galinstan droplet was at the electrode (2). $\overline{{Z }_{2}}$ shows the change in the variable resistor as an impedance. As a control experiment, the impedance change rate $\overline{{Z }_{3}}=({Z}_{3}-{Z}_{1})/{Z}_{1}$ of electrode (3) relative to electrode (1) is measured when a Galinstan droplet is at electrode (3). $\overline{{Z }_{3}}$ enables us to measure how much the signal value of the sensor has changed. Figure 6 shows the impedance change rates ($\overline{{Z }_{2}}$ and $\overline{{Z }_{3}}$) and as a function of the resistance of the variable resistor connected to electrode (2). We can see that when the resistance of the variable resistor connected to the electrode (2) changes, the rate of change of impedance $\overline{{Z }_{2}}$ changes. This makes it possible to identify how much the signal value of the sensor has changed. The rate of change of impedance $\overline{{Z }_{3}}$ for the control experiment is close to zero, regardless of the resistance value of the variable resistor. Since both the calibration resistance and the reference resistance are 1000 Ω, this indicates that the value of the resistance for reference can be read regardless of the other electrode. These results show that it is possible to take out the signal of each electrode independently and that it can be realized as the mechanism toward a multiplexer.

## Discussion

In the experiment, a Galinstan droplet is introduced between the electrodes connected to the resistance for the calibration, the variable (signal) resistor, and the resistance for the reference, corresponding to electrodes (1), (2), and (3) in Fig. 4, respectively. When the resistance of the variable resistor connected to electrode (2) decreases, the impedance changes from the initial value (Fig. 4). The impedance with a Galinstan droplet at electrode (2) is lower than the impedance with a Galinstan droplet at electrode (1) connected to the resistance for the calibration. In the circuit diagram in Fig. 3b, a series of circuits of a capacitor (electrode pair) and resistance are connected in parallel. We define the impedances of the circuits composed of electrode (1), electrode (2), and electrode (3) as *Z*_*RC*1_, *Z*_*RC*2_, and *Z*_*RC*3_, respectively. The impedance of the entire circuit, *Z*_*Total*_, is given as follows.

1 $${{Z}}_{{{Total}} \, }= \frac{1}{\frac{1}{{{Z}}_{{{RC}}{1}}}{+}\frac{1}{{{Z}}_{{{RC}}{2}}}{+}\frac{1}{{{Z}}_{{{RC}}{3}}}}.$$

Equations (2)–(4) show *Z*_*RC1*_, *Z*_*RC2*_, and *Z*_*RC3*_ using the resistance values (*R*_1_, *R*_2_, and *R*_3_) and capacitance (*C*_1_, *C*_2_, and *C*_3_), respectively.

2 $${{Z}}_{{{RC}}{1}}{ = }{{R}}_{1}{+}\frac{1}{{i\omega}{{C}}_{1}}{,}$$

3 $${{Z}}_{{{RC}}{2}}{ = }{{R}}_{2}{+}\frac{1}{{i\omega}{{C}}_{2}}{,}$$

4 $${{Z}}_{{{RC}}{2}}{ = }{{R}}_{3}{+}\frac{1}{{i\omega}{{C}}_{3}}.$$

When the Galinstan droplet replaces silicone oil between electrodes, the gap of electrodes decreases, which causes the increment of the capacitance. This behavior agrees with the experimental result shown in Fig. 2. Then, the decrement of the capacitance decreases the second term of Eqs. (2)–(4) and as a result, *Z*_*RC*1_, *Z*_*RC*2_, and *Z*_*RC*3_ also decrease. Thus, *Z*_total_ in Eq. (1) decreases, which agrees with the experimental result shown in Figs. 5 and 6.

In our experiment, the calibration resistance and the reference resistance are equal (*R*_1_ = *R*_3_ = 1000 Ω). When the Galinstan droplet is sandwiched by electrode (2), *C*_1_ equals *C*_3_ (*C*_1_ = *C*_3_). First, we consider the change in total impedance with decreasing resistance of the variable resistor. *Z*_*RC*2_ in Eq. (2) decreases with decreasing *R*_2_. Therefore, 1/*Z*_*RC*2_ on the right side of Eq. (1) becomes larger. Since *Z*_*RC*1_ and *Z*_*RC*3_ do not depend on the resistance value, *Z*_*Total*_ decreases. These results suggest that decreasing *R*_2_ causes a decrease in *Z*_*Total*_ independent of the position of the Galinstan droplet, which agrees with our experimental results.

Next, we discuss the impedance change concerning the position of the Galinstan droplet. For simplicity, we use the combined impedance of *Z*_*RC*1_ and *Z*_*RC3*_, ${Z}_{RC13}=A+Bi$, where *A* and *B* are the real and imaginary parts of the combined impedance, respectively. Substituting Eq. (1) together with Eq. (3) for ${Z}_{RC2}$, the real part of the total impedance, *Z*_*ReTotal*_ is given as follows,

5 $$Z_{{ReTotal}} = \frac{{\frac{A}{{{\omega ^{2}} {C_{2} ^{2}} }} + B^{2} R_{2} + AR_{2} ^{2} + A^{2} R_{2} }}{{\left( {A + R_{2} } \right)^{2} + \left( {B + \frac{1}{{{\omega} C_{2} }}} \right)^{2} }}.$$

The capacitance of one electrode pair, *C* is approximately 7 pF, and the measurement frequency of the LCR meter is 1 MHz. Therefore, we obtain $1/\omega C\gg R$. *B* is the imaginary part of the impedance synthesized from the parallel circuits of *Z*_*RC*1_ and *Z*_*RC*3_. Since there are no Galinstan droplets on electrode (1) and electrode (3), *C* = *C*_1_ = *C*_3._ Then, by ignoring the *R* term, we obtain $\left|B\right|=1/2\omega C$. *Z*_*ReTotal*_ can be approximated as follows:

6 $$Z_{{ReTotal}} \approx \frac{{\frac{A}{{{\omega^{2}} {C_{2}^{2}} }} + \frac{{R_{2} }}{{4{\omega^{2}} {C^{2}} }}}}{{\left( {\frac{1}{{2{\omega} C}} + \frac{1}{{{\omega} C_{2} }}} \right)^{2} }}.$$

For a Galinstan droplet at electrode (2), the capacitance *C*_2_ becomes larger than *C*. Hence, $1/\omega C>1/{\omega C}_{2}$ and the *R*_2_ term in Eq. (6) is dominant in *Z*_*ReTotal*_. When the Galinstan droplet is located at electrodes (1) or (3), the influence of *R*_1_ and *R*_3_ is expected to be dominant. In other words, the total impedance depends mainly on the resistance of the sensor connected to the electrode sandwiching the Galinstan droplet. Therefore, as the resistance of the variable resistor decreases, *Z*_*ReRotal*_ decreases, and the amount of decrease in *Z*_*ReTotal*_ with the Galinstan droplet at electrode (2) becomes the largest, which agrees with the experimental result shown in Fig. 6.

As in the control experiment in Fig. 6, we decrease the resistance of the variable resistor connected to electrode (2) and measure the impedance with the Galinstan droplet at electrode (3). In this control experiment, Eq. (6) shows that the impedance value with the Galinstan droplet at electrode (3) should be the same as the impedance at electrode (1) because the effect of *R*_3_ connected to electrode (3) is dominant. This indicates that the signal from the reference electrode does not depend on the resistance of the variable resistor and that it is possible to extract the signal independently for each sensor connected to the electrodes.

Next, within our proposed mechanism, we control the position of Galinstan droplets in the microchannel by applied pressure. To fix the position of Galinstan droplets in the microchannel without pressure, the diameter of Galinstan droplets, *a*, must be larger than that of the microchannel. Since the diameter of our microchannel is 0.5 mm, *a* must be larger than 0.5 mm. In addition, the measurement resolution of capacitance requires the minimum length of the Galinstan droplet. The insertion of Galinstan droplets should cause an observable change in capacitance. Let us discuss the minimum length, *L*_min_, based on the measurement resolution of the capacitance. In the experiment, we used the Galinstan droplet with a length of 10 mm and obtained the capacitance change of 0.07 pF. The capacitance change is proportional to the length of Galinstan droplets. Considering Taking into account the resolution of the LCR meter (~ 0.01 pF), *L*_min_ ~ 1.4 mm.

Here, the gap of the electrodes was set to be 1 mm. Besides, the microfabrication technique should decrease the gap of the electrodes, which causes the increment of the capacitance. Thus, we can obtain the capacitance change even with short Galinstan droplets. Simultaneously, the microfabrication of the channel should decrease the diameter of Galinstan droplets. Thus, the measurement resolution does not restrict the minimum size of Galinstan droplets can be small. On the other hand, the microfabrication of the microchannel restricts the switching speed of the multiplexer. Since the flow rate, according to the previous work^24^, is proportional to *a*^4^, the microfabrication decreases the switching speed of Galinstan droplets. For these reasons, the required switching speed determines the minimum diameter of the microchannel.

## Materials and methods

### The structure for the mechanism toward a multiplexer

**Figure 7:**
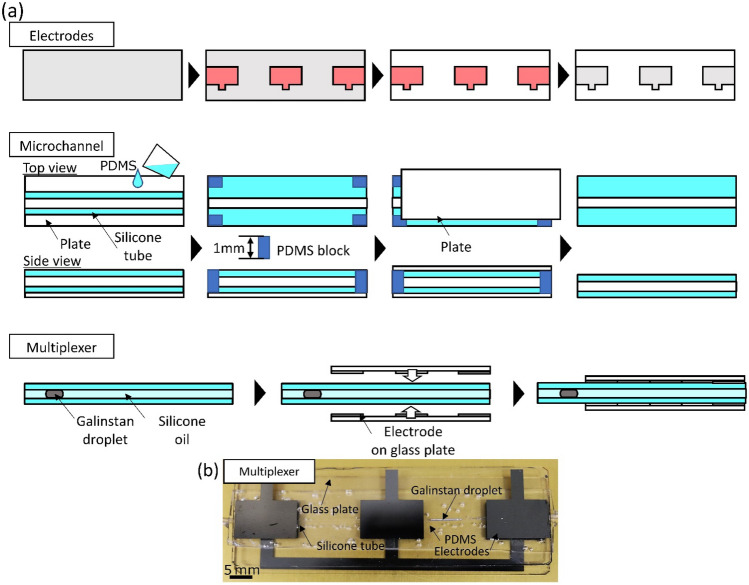
Fabrication process for microchannel sandwiched by pairs of electrodes. (**a**) Fabrication process of the multiplexer. We fabricated the electrodes and the microchannel, separately and sandwiched the microchannel between the electrodes. The gap and electrodes and the thickness of the microchannel is 1 mm. (**b**) A perspective photograph of the multiplexer. The scale bar indicates 5 mm.

We integrated flexible channels and electrodes patterned on glass substrates to construct the test structure. Figure 7a shows the fabrication process of the microchannel sandwiched by pairs of electrodes. To prepare the electrodes, we deposited a chromium layer with a thickness of 2.0 Å on a glass plate. Then, a photoresist (OFPR-800LB, Tokyo Ohka Kogyo Co.) layer is patterned on the chromium layer using photolithography. The Cr layer is patterned by the wet-etching technique. We obtained patterned chromium electrodes using photolithography. The size of the 2^3^ pairs of electrodes (Fig. 2) was 10 mm × 10 mm and the electrodes were connected. The distance between adjacent electrodes was 10 mm. The size of the three pairs of electrodes (Fig. 3) was 10 mm × 15 mm, and the distance between adjacent electrodes was 15 mm. In principle, the wide of the electrodes does not need to be smaller than the diameter of the microchannel. On the other hand, small electrodes cause difficulty of alignment and the unstable measurement value of the capacitance. Since we aim not to optimize the fabrication process but to investigate the multiplexer mechanism, we designed our devices with a margin to make the fabrication process easy. In addition to the electrode-patterned glass plate, we patterned the wiring to connect the electrodes. The microchannel was sandwiched by the two glass plates so that the top and bottom electrodes overlapped.

Next, we fabricate the microchannel. We used a silicone tube (ASONE) with an inner diameter of Φ 0.5 mm and an outer diameter of Φ 1 mm as the microchannel. Then, we enclosed the tube with PDMS (polydimethylpolysiloxane, DuPont Toray Specialty Materials K.K.) composed of the same silicone material as the microchannel. The thickness of PDMS was 1 mm. We use PDMS because (1) PDMS was used in previous work, and (2) the dielectric constant of PDMS is approximately equal to that of silicone oil. Furthermore, PDMS can be easily formed into microstructures through molding technology. PDMS is widely used in structures for lab-on-a-chip. We added PDMS to a silicone tube sandwiched between two glass plates whose gap is adjusted to be 1 mm with PDMS blocks. After the annealing process of PDMS, we removed the glass substrates. A Galinstan droplet (eutectic gallium indium stannum, Zairyo-ya.com) and silicone oil (Shin-Etsu Chemical Co., Ltd.) were injected into the microchannel. The length of the Galinstan droplet was 5 mm for 2^3^ pairs of electrode arrays and 10 mm for 3 pairs of electrodes. Here, we set the length of the Galinstan droplet shorter than the width of the adjacent electrodes. Then, we sandwiched the microchannel between electrode patterned substrates and obtained the microchannel device.

Figure 7b shows the perspective view of the fabricated microchannel sandwiched by 3 pairs of electrodes. We connected the electrodes on the upper substrate and LCR meter (Agilent, 4284A) directly. On the other hand, the electrodes on the lower substrate were connected with resistors which are regarded as sensors. Galinstan droplets in the cylindrical microchannel can be transported smoothly. For example, previous work reported the smooth transportation of Galinstan droplets in the microchannel fabricated using a molding method^24^. We can apply these microfabrication techniques to our multiplexer system easily. Thus, Galinstan droplets should serve as a switching mechanism of multiplexer even for complex channel geometry.

### Measurement method of capacitance and impedance

To control the position of the Galinstan droplet in the microchannel, we manually applied pressure with a syringe filled with silicone oil. In addition, the other end of the microchannel was placed in silicone oil so that the microchannel was always filled with silicone oil. In the experiment, we applied pressure and confirmed the Galinstan droplet introduced between the pair of electrodes. To perform a stable measurement, we stopped applying pressure for 10 s. Then, we applied pressure again and stopped the Galinstan droplet between the adjacent pairs of electrodes. We measured the capacitance and impedance when repeating this process. We used an LCR meter to measure the capacitance and impedance. The frequency and voltage in the measurement were 1 MHz and 1 V, respectively.

In the experiment for measuring the capacitance, we used 2^3^ pairs of electrodes and a microchannel sandwiched between them. We controlled the position of the Galinstan droplet. Then, we connected both the top and bottom wires to the LCR meter. We moved the Galinstan droplet and measured the capacitance.

In the experiment for measuring the impedance, the three pairs of electrodes were connected to the resistance elements, as shown in Fig. 3. The electrodes of both sides (electrodes (1) and (3) in Fig. 3) were connected to a 1000 Ω resistor, and the electrode of the center (electrode (2) in Fig. 3) was connected to a variable resistor. The wiring connected the three resistors and the LCR meter. We measured the synthetic impedance and the phase. Based on the synthetic impedance and the phase, we calculated the real and imaginary parts of the impedance.

## Conclusion

In this paper, we succeeded in detecting the signal from multiple sensors integrated into the microchannel using position control of the Galinstan droplets in the microchannel. We introduced Galinstan droplets into the microchannel sandwiched between electrode pairs. The electrode pairs can be regarded as capacitors. Then, the insertion of the Galinstan droplet increases the capacitance of the electrode pair. We connected the electrode pairs and resistive sensors and investigated the relationship between signals and the position of the introduced Galinstan droplet. As a result, we showed the selective acquisition of the signal from the sensor connecting with the electrode pair between which the Galinstan droplets are inserted. This suggests that we can detect the signal from the multiple sensors selectively within our multiplexer mechanism. LMD shuttling in the microchannel should apply to the multiplexer without complex wire networks. In this study, for simplicity, we used a test structure composed of thin chromium electrodes patterned on glass substrates. As prospects, it is possible to introduce flexible electrodes or flexible substrates for electrodes in accordance with requirement for application of flexible structure, such as catheter and endoscope. For example, we reported the integration of a flexible temperature sensor with a soft microactuator made of PDMS^25^. Thermocouples using a pair of dissimilar metals or alloys were integrated into the soft structure. These technologies enable us to apply the multiplex mechanism to flexible systems.

## Data Availability

All data generated or analyzed during this study are included in this published article.
